# Potential role of CBX7 in regulating pluripotency of adult human pluripotent-like olfactory stem cells in stroke model

**DOI:** 10.1038/s41419-018-0519-8

**Published:** 2018-05-02

**Authors:** Jia-Rong Fan, Hsu-Tung Lee, Wei Lee, Chen-Huan Lin, Chun Y. Hsu, Chia-Hung Hsieh, Woei-Cherng Shyu

**Affiliations:** 1 Translational Medicine Research Center, and Department of Neurology, China Medical University Hospital, Taichung, 40440 Taiwan; 2 Department of Neurosurgery, Taichung Veterans General Hospital, Taichung, 40421 Taiwan; 3 Graduate Institute of Medical Sciences, National Defense Medical Center, Taipei, Taiwan; 4 Graduate Institute of Biomedical Science, China Medical University Hospital, Taichung, 40440 Taiwan; 5 Department of Occupational Therapy, Asia University, Taichung, Taiwan

## Abstract

The adult olfactory mucosa, a highly regenerative tissue with unique life-long neurogenesis ability, is thought to harbor a naïve yet tightly controlled stem cell population. It will provide unique benefits in various stem cell-based therapies, such as stroke treatment. Here, we identified a subpopulation of adult pluripotent-like olfactory stem cells (APOSCs), which were modulated by an epigenetic repressor of CBX7. APOSCs form a floating sphere, express pluripotency markers Nanog, Oct-4, Sox-2, and SSEA-4 and show alkaline phosphatase activity. In addition, APOSCs display self-renewal and a pluripotent potential to differentiate into all three germ layers. Moreover, APOSCs coexpress pluripotency markers with CBX7. Within their natural niche, APOSCs from CBX7^+/+^ mice responded promptly to either spontaneous or injury-induced tissue regeneration. However, APOSCs from CBX7^−/−^ mice manifested an impaired self-renewal and differentiation potential. Similarly, in vitro-cultivated CBX7^−/−^ APOSCs underwent premature senescence, whereas CBX7^+/+^ APOSCs still actively divided, indicating that CBX7 is required for the self-renewal of APOSCs. Intracerebral implantation of APOSCs improved the stroke-mediated neurological dysfunction in rodents. These findings indicate that CBX7 plays a critical role in the regenerative properties of APOSCs and indicate the safety and feasibility of implantation of autologous APOSCs in stroke treatment.

## Introduction

The holy grail of adult stem cell research is to discover pluripotent-like stem cells among adult normal tissues^1,2^. Accumulating evidence revealed the presence of embryonic stem cells (ESC)-mimicking stem cells in various adult mammalian craniofacial compartments^3,4^. For example, stem cells isolated from dental pulp^5^, oral mucosa^6^, and respiratory mucosa^7^ behave as pluripotent self-renewing cells that carry ESCs markers and can differentiate into multiple lineages. Accordingly, we seek to identify novel pluripotent-like adult stem cells in another craniofacial compartment: the olfactory mucosa, a highly regenerative tissue with life-long neurogenesis capacity.

The olfactory mucosa is composed primarily of olfactory receptor neurons (ORN) and sustentacular cells (Sus)^8^, underlined with the basal membrane (BM) and lamina propria (LP). Upon extensive tissue injuries, normally quiescent stem cells can transiently proliferate to reconstitute ORN^9^. Numerous stem cell populations have been discovered within the olfactory mucosa, such as horizontal basal cells (HBC) and globose basal cells (GBC), which reside in the BM; olfactory ensheathing cells (OECs) and olfactory ensheathing mucosa mesenchymal stem cells (OE-MSCs), which reside in the LP^3,10^. There is another multipotent population originated from the murine olfactory mucosa, which could generate numerous cell types when transplanted into the chicken embryo^11^. However, whether the human adult olfactory mucosa harbors a naive stem cell population that possesses pluripotency-related markers and the ability to differentiate into the three germ layers has not been demonstrated.

Little is known about the molecular mechanisms that govern olfactory stem cells in an undifferentiated state, and drive their self-renewal when tissue damage occurs. CBX7 is a focus of research because it is essential for the maintenance of embryonic stem cells (ESCs)^12,13^ and several adult stem cell types, including central neural^14,15^, hematopoietic^16^. As a key subunit of PRC1 (polycomb repressive complex 1), CBX7 is required for maintaining other stem cells by preventing cellular senescence, Until now, whether CBX7 is expressed in the adult olfactory mucosa and its putative role in regulation of adult olfactory stem cells remain unexplored.

Acute ischemic stroke, which is caused by occlusion of a cerebral artery, results in damage to neurons, astrocytes, and endothelial cells. Therefore, various preclinical adult stem cell therapies, including a transplant of bone marrow stem cells, umbilical cord blood cells, or adipose pluripotent stromal cells, are under development for stroke treatment^17,18^. It is intriguing to determine whether adult olfactory stem cells also hold a potential for stroke treatment. Here, we isolated a new subpopulation of **a**dult **p**luripotent-like **o**lfactory **s**tem **c**ells (APOSCs), which carry ESCs markers and harbor a significant three-germ layer differentiation potential, from both human and mouse olfactory mucosa. Moreover, knockout experiments show that CBX7 modulates the self-renewal and senescence in APOSCs.

## Results

### Isolation of a pluripotent-marker-expressing population of APOSCs

In the search for pluripotent-like cells from APOSCs, some typical ESCs characteristics serve as critical criteria^19^. First, the expression of (i) key transcription factors, such as Nanog, Sox-2, and Oct-4^20^, which are essential for the developing blastocyst; or (ii) cell surface glycosphingolipids present on undifferentiated human ESCs, such as stage-specific embryonic antigen SSEA-3 and SSEA-4^21,22^, should be demonstrated in adult-tissue-derived stem cells. Second, “plasticity” experiments should show a contribution of adult stem cells to generation of tissues originated from all three germ layers.

**Fig. 1:**
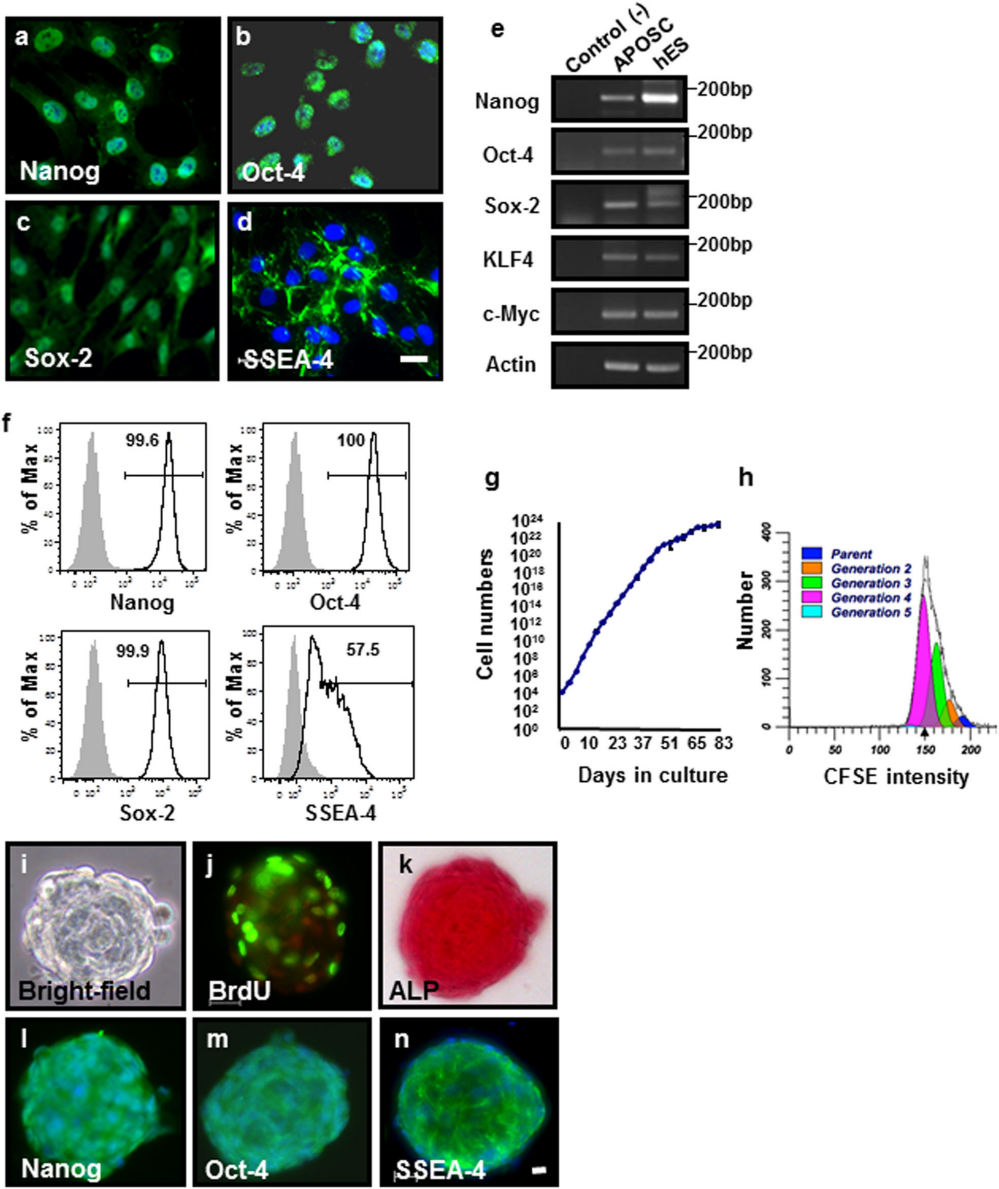
Expression of pluripotency-related markers in APOSCs isolated from the human olfactory mucosa. **a–d** Immunocytochemistry (green) showed expression of Nanog (**a**), Oct-4 (**b**), Sox-2 (**c**), and SSEA-4 (**d**) proteins in isolated human APOSCs. Cell nuclei were stained with DAPI (blue). **e** RT-PCR analyses of isolated human APOSCs. Human ES (hES) cells served as the positive control (twofold diluent cDNA). Templates without reverse transcription served as the negative control (-). **f** Flow cytometric analysis of isolated human APOSCs for Nanog, Oct-4, Sox-2, and SSEA-4 expression using specific antibodies (open area curve) or respective isotype control antibodies (filled area curve). Proportions of positive cells (%) are shown on upper right of each panel as compared to controls. **g** Expansion capacity of long-term cultivated human APOSCs. Shown are averages in duplicates of APOSCs. **h** Flow cytometric analysis of APOSCs distribution according to their CFSE fluorescence after 4-day cultivation. Peaks represent the number of cells corresponding to each of the subsequent daughter generations (number of cells analyzed: 10^4^). **i–n** Characterization of human APOSC-formed primary spheres. Morphology (**i**) of a sphere under a bright-field microscope. A BrdU-labeled (green, **j**) sphere. Cell nuclei were stained with PI (red). ALP staining (red, **k**) of an APOSCs sphere. Immunocytochemistry (green) and DAPI staining (blue) of APOSCs spheres for Nanog (**l**), Oct-4 (**m**), and SSEA-4 (**n**) expression. Scale bar, 20 μm

We first sought to determine the existence of an adult olfactory cell population (APOSCs) that expresses ESCs markers. Human APOSCs showed the ability to migrate from the dissociated olfactory mucosal tissues (i.e., explant) and formed small groups of cells in a confluent monolayer during 2−3 weeks of culturing. Isolated APOSCs were then evaluated for expression of pluripotency markers. Nanog, Oct-4, and Sox-2 were expressed in APOSCs as shown by immunocytochemistry (Fig. 1a–c), reverse transcription polymerase chain reaction (RT-PCR) (Fig. 1e), and flow cytometry (Fig. 1f). Accordingly, c-Myc and KLF-4, which contributed to generation of induced pluripotent stem (iPS) cells^23^, were also expressed in APOSCs (Fig. 1e). Additionally, the presence of SSEA-4 on APOSCs surface indicated a more primitive state of these cells (Fig. 1d). Flow cytometric analysis of APOSCs cultures derived from 6 donors, at various passages (p2–p14) revealed that 52.9% ± 19.3% (*n* = 6 donors) of these cell populations were positive for SSEA-4 (Fig. 1f). Even the more primitive-stage marker, SSEA-3, was detected in a small portion of APOSCs (varying from 0.1% to 5% among six donors). Immunocytochemistry also confirmed nuclear expression of Nanog and Oct-4 in APOSC-derived from these 6 donors (data not shown).

To analyze the proliferation ability of in vitro-cultivated APOSCs, long-term expansion of APOSCs was achieved. They proliferated exponentially for 48 days (17 passages), and grew slowly after 83 days (25 passages) (Fig. 1g). The calculated doubling time of APOSCs was 20.9 ± 3.9 h (donor 1, average of passages 3 to 17), or 25.3 ± 6.7 h (donor 2, average of passages 5 to 19). Such active growth phenotype of APOSCs was further substantiated by tracking cell division by pulse labeling of the cells with CFSE (carboxyfluorescein succinimidyl ester), whose fluorescence decreased precisely twofold at each successive cell generation. During the 96-h tracking period, 50.9% of APOSCs had completed up to three divisions (Fig. 1h), and 32.7% of them completed two cell divisions (green area in Fig. 1h). This analysis showed that the cell cycle kinetic of APOSCs was equivalent to that of human ESCs, iPS cells (doubling time between 24 to 48 h)^23,24^ and exceeded that of some adult multipotent stem cells (doubling times varying from 30 to 72 h)^6,7,25^. To demonstrate the negative control data, differentiated APOSCs show no expression of CD45 evaluated by the immunocytochemistry (ICC) (Supplementary Fig. S1h).

Similar characterization results were obtained when mouse APOSCs were isolated from a mouse olfactory mucosa explant, except that successful culture/expansion of mouse APOSCs required additional epidermal growth factor (EGF) supplement. Mouse APOSCs expressed Nanog, Oct-4, Sox-2 (Supplementary Fig. S1a–c), KLF-4 and c-Myc (Supplementary Fig. S1i) within the nucleus. The doubling time of mouse APOSCs was 24.6 ± 2.0 h. Moreover, representative differentiation markers of three germ layers (Ectoderm: Tuj-1; Mesoderm: α-SMA; and endoderm: albumin) revealed no immunoreactivity in non-differentiated APOSCs by ICC (Supplementary Fig. S1j). However, lower telomerase activity of APOSCs (both CBX7^+/+^ and Cbx7^−/−^ cells) was observed compared to the ESCs (Supplementary Fig. S1k). In contrast to the prominent teratoma formation after ESCs inoculation to nude mice, there was no teratoma mass after the human APOSCs inoculation (Supplementary Fig. S1l).

### Three-dimensional growing spheres of APOSCs

One of the distinct characteristics of stem cells is their ability to form spheres when subjected to a three-dimensional, natural-niche-mimicking environment^26–28^. Therefore, aside from adhesive growth, APOSCs were cultivated under suspension culture conditions to test whether they could maintain their stemness as spheres. Human APOSCs effectively formed compact floating spheres in suspension culture (Fig. 1i). Furthermore, APOSCs spheres were tested whether they possess self-renewing ability. The incorporation of BrdU in primarily formed APOSCs spheres revealed their persistent entry into the S phase (Fig. 1j). The cell proliferation marker, Ki67, was also abundantly expressed in primary APOSCs spheres (data not shown). Subsequently, primary APOSCs spheres (2-day-cultivated) were dissociated into single cells and returned to suspension culture. After 2 days, 50% of the dissociated cells survived and secondary APOSCs spheres arose in suspension culture. The diameter of secondary APOSCs spheres cultivated over 9 days was measured to show an increase of the mean diameter of these spheres from 59 to 81 m. Moreover, the APOSC spheres tested positive for Nanog (Fig. 1l), Oct-4 (Fig. 1m), and SSEA-4 (Fig. 1n). As in ESCs, alkaline phosphatase (ALP) activity was detected in APOSCs spheres (Fig. 1k). Similar characteristics were observed in mouse APOSCs spheres (Fig. S1d–g).

### The multipotent differentiation potential of APOSCs in vitro

To analyze the differentiation ability of APOSCs, certain growth factor-based induction systems were used to guide human APOSCs to differentiate into the ectoderm (neural cells), mesoderm (adipocytes, osteoblasts, chondrocytes, and endothelial cells) or endoderm (hepatocytes).

**Fig. 2:**
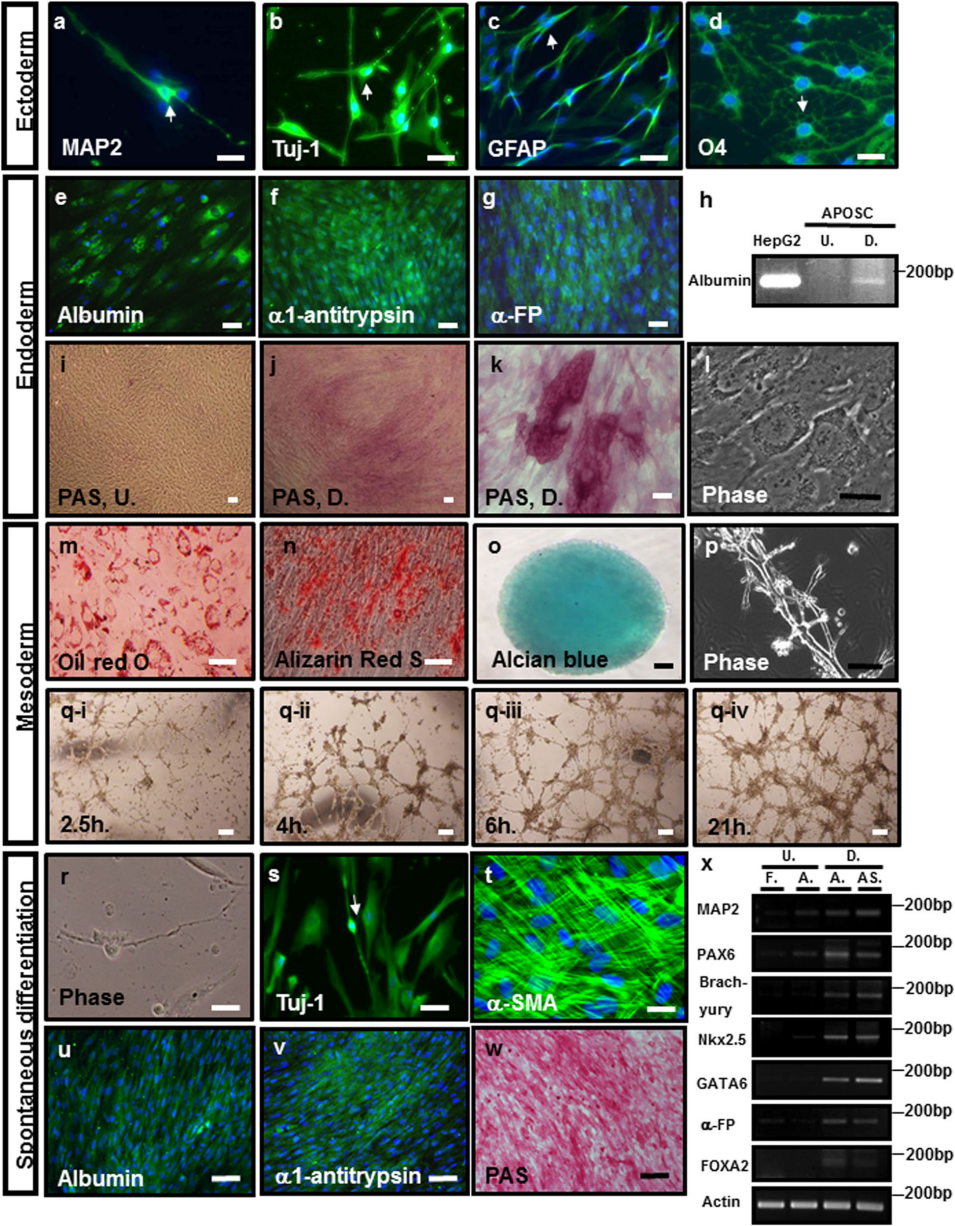
Induced and spontaneous differentiation of APOSCs into cell lineages of three germ layers in vitro. **a–d** Induced ectoderm differentiation. Various neuron markers and morphological features exhibited by APOSCs after differentiation. Immunocytochemistry showed MAP2, Tuj-1 (green, **a** and **b**), GFAP (green, **c**), and O4 (green, **d**) expression in cells. Cell nuclei were stained with DAPI (blue). **e–l** Induced endoderm differentiation. Immunocytochemistry (green) of APOSC-derived hepatocytes showed positive signals of albumin (**e**), α-antitrypsin (**f**), and α-FP (**g**). Cell nuclei were stained with DAPI (blue). **h** RT-PCR analysis showed albumin expression in differentiated APOSCs (**d**) but not in undifferentiated APOSCs (U). Human liver carcinoma cell line, HepG2, served as a positive control. Differentiated hepatocytes expressed glycogen deposits as shown by PAS stain (staining shown in **j** and **k**) and the phase contrast micrograph (**l**). Negative PAS staining of undifferentiated APOSCs (in **i**). Scale bar, 20 μm. **m–q** Induced adipocyte differentiation of APOSCs (**m**). After differentiation, APOSCs contained lipid droplets according to staining with oil red O. APOSCs were induced to differentiate into osteoblasts expressing osteocalcin according to staining with Alizarin Red S (**n**). Induced differentiation of APOSCs into chondrocytes (**o**). Alcian Blue staining indicated proteoglycans synthesized by chondrocytes generated from APOSCs. Tube formation ability of APOSCs (**p**–**q**). Spontaneously, APOSCs could differentiate into endothelial cells, as verified by their tube-forming ability in this assay (in **p**). Dynamics of the behavior of VEGF-stimulated APOSCs after plating on Matrigel. The photos were taken at 2.5 h (**q**–i), 4 h (**q**-ii), 6 h (**q**-iii), or 21 h (**q**-iv) after plating of APOSCs for the tube formation assay. Note that tubes were matured at 6 h and broken apart at 21 h. **r–x** Spontaneous differentiation of human APOSCs into three germ lineages. Representative images of APOSC-derived neurons (**r**, bright-field micrograph and **s**, immunocytochemistry for Tuj-1, green), α-SMA-positive cells (**t**), albumin- (**u**), and α1-antitrypsin-positive (**v**) cells (immunocytochemistry, green). Cell nuclei were stained with DAPI (blue). **w** PAS staining of spontaneously differentiated APOSCs. **x** RT-PCR analyses of various differentiation markers of three germ layers. F.: human fibroblasts, A.: human APOSCs, AS.: APOSCs-Spheres, U: undifferentiated cells, D.: differentiated cells. Scale bar, 20 μm

Induced neuronal differentiation resulted in a high frequency (over 85%) of cells positive for the neuronal marker microtubule-associated protein 2 (MAP2) as well as β-III-tubulin (Tuj-1) (Fig. 2a, b), astrocyte marker of glial fibrillary acidic protein (GFAP, Fig. 2c) and oligodendrocyte marker of O4 (Fig. 2d). These cells showed neuronal morphology, including long bipolar thread-like morphology (Fig. 2b, resembling developing ORNs), multipolar morphology, as well as branching (Fig. 2c, arrow), webbed (Fig. 2c, d, arrow) or beaded (Fig. 2a, arrow) axon-like structures.

Hepatocyte induction produced cells positive for hepatocyte-specific genes, including albumin (Fig. 2e, h), α1-antitrypsin (Fig. 2f), and human α-fetoprotein (α-FP, Fig. 2g). To test for hepatocellular metabolic functions, polyglycans were stained by the periodic acid schiff (PAS) method, which indicated glycogen storage within the cytoplasm of differentiated APOSCs (Fig. 2j, k), but not within undifferentiated APOSCs (Fig. 2i).

Adipogenic differentiation of APOSCs could be identified via Oil Red O staining, as an indicator for intracellular lipid accumulation (Fig. 2m). Osteogenesis in APOSCs was detected via Alizarin Red S staining to show calcium mineralization (Fig. 2n). Chondrogenesis was detected via Alcian Blue staining, the indicator of proteoglycans synthesized by differentiated APOSCs (Fig. 2o). Moreover, to demonstrate that APOSCs could be directed to differentiate into endothelial cells, APOSCs were exposed to angiogenic factors VEGF, bFGF, and heparin to generate endothelial cells. In the tube formation assay (as an in vitro angiogenesis model), the stimulated APOSCs formed capillary-like tube structures efficiently (Fig. 2q). APOSCs initially attached, migrated toward each other within 2–4 h (Fig. 2q-i, ii) and then formed capillary-like tubes, which matured by ~6 h (Fig. 2q-iii). After 21 h, the tubes detached from the matrix and broke apart (Fig. 2q-iv). Such tube formation kinetics are typical for endothelial cells^29^. Without beforehand VEGF-induction, APOSCs could also form tubes with a lumen in a tube formation assay (Fig. 2p), although with delayed kinetics (tube growth began at 48 h).

Therefore, to test whether APOSCs could spontaneously differentiate into three germ layer-derived cells, without any growth factors direction. APOSCs or primary spheres were transferred onto gelatin-coated dishes for 15 days of culture. Neuron-like cells were observed in the spontaneously differentiated population (Fig. 2r). Immunocytochemistry reveled cells positive for Tuj-1 (an ectodermic marker, Fig. 2s), α-smooth muscle actin (α-SMA; a mesodermic marker, Fig. 2t), albumin and α1-antitrypsin (endodermic markers, Fig. 2u, v). A PAS showed the glycogen deposition in cells (indicating endodermic hepatocytes, Fig. 2w). RT-PCR of gelatin-grown APOSCs/or APOSCs spheres confirmed that these cells increasingly expressed ectodermic markers MAP2 and PAX6, mesodermic markers Brachyury and Nkx2.5, as well as endodermic markers GATA6, α-FP, and FOXA2 (Fig. 2x).

### In vivo differentiation of APOSCs

**Fig. 3:**
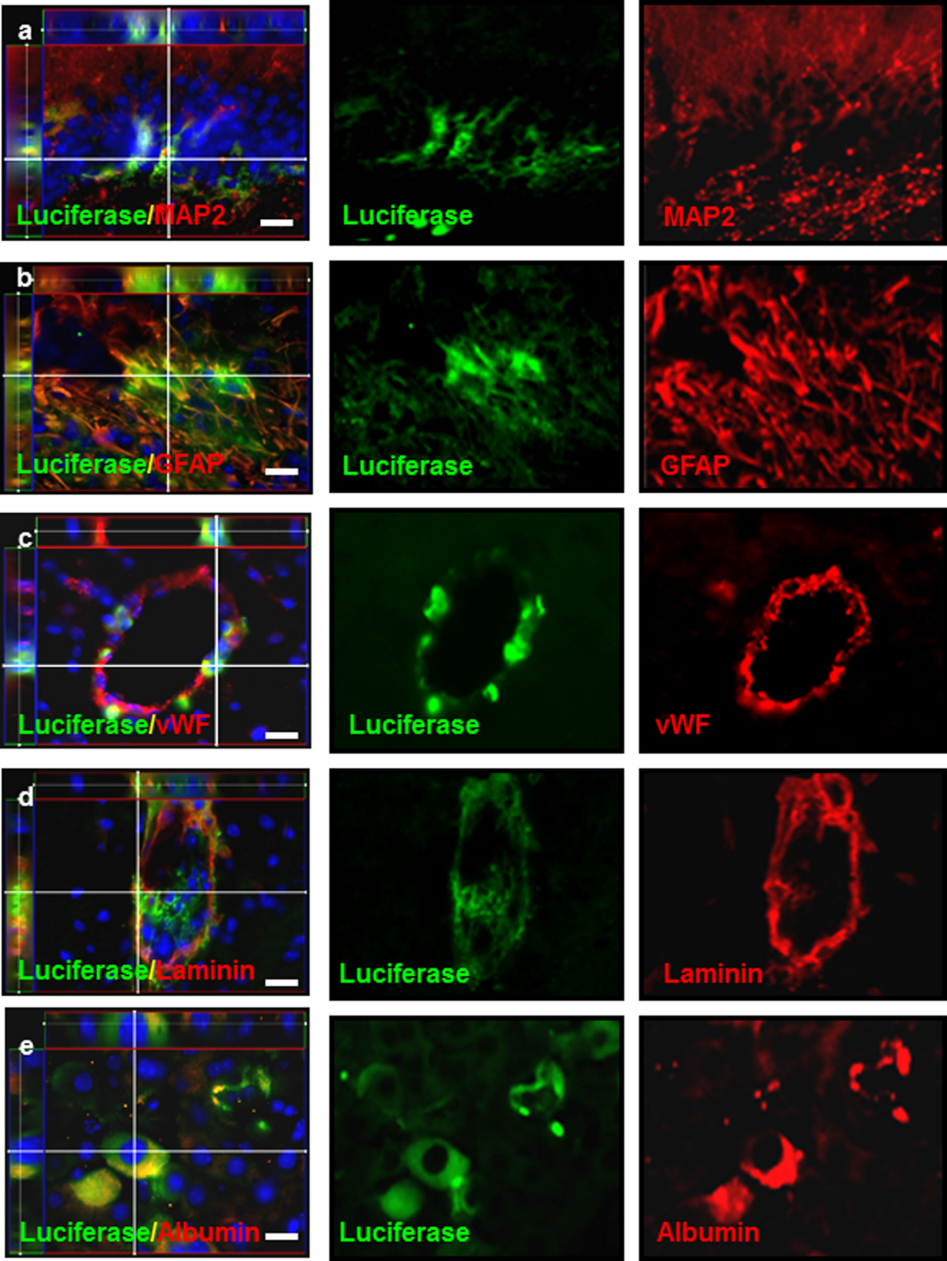
In vivo differentiation of human APOSCs in mice. **a–d** Intracerebrally transplanted hAPOSCs-Luc (luciferase^+^, green) expressed MAP2 (**a**, red), GFAP (**b**, red), vWF (**c**, red), and laminin (**d**, red). **e** Differentiation of hAPOSCs-luc to hepatocytes in mouse liver. Some of the transplanted hAPOSCs-Luc (luciferase^+^, green) expressed human albumin (red). The enlarged 3D images (left in each panel) revealed cells coexpressing luciferase with specific cell type markers. Cell nuclei were stained with DAPI (blue). Scale bar, 20 μm

Furthermore, to determine whether hAPOSCs-Luc could differentiate into neurons, glial cells, or endothelial cells in the mouse brain after intracerebral hAPOSC-Luc injection into mice under cerebral ischemia. After 4 weeks, exogenous transplanted hAPOSCs-Luc engrafted into the penumbra area, lateral ventricle (LV), and hippocampal dentate gyrus (DG) of the ischemic hemisphere. Immunofluorescence colocalization results showed that some luciferase-labeled cells coexpressed MAP2 (Fig. 3a, in DG) or GFAP (Fig. 3b, in penumbra area). We also found that cells around the lumen of blood vessels coexpressed luciferase with endothelial cell markers, vWF or laminin (Fig. 3c, d). Semiquantitatively, transplanted cells were engrafted to differentiatie into MAP2^+^ neurons (4.2 ± 0.8%), GFAP^+^ glial cells (7.6 ± 0.3%), and vWF^+^ endothelial cells (10.4 ± 0.5%). Similarly, the transplanted mAPOSC-GFP (transgenic GFP-mice-derived mouse APOSCs) also migrated to the penumbra area, differentiated into neurons (MAP2^+^), neural progenitor cells (Nestin^+^) or endothelial cells (vWF^+^, Laminin^+^) (Supplementary Fig. S2).

To determine whether APOSCs could differentiate into hepatocytes in vivo, hAPOSC-Luc were transcutaneously injected into a neonatal mouse liver^30^, a proliferative environment beneficial for the putative expansion of engrafted APOSCs. Six weeks after transplantation, a few hepatocytes coexpressing luciferase and human albumin could be detected across mouse livers (Fig. 3e).

### The basal layer of adult human and murine olfactory epithelia as the origin of APOSCs

**Fig. 4:**
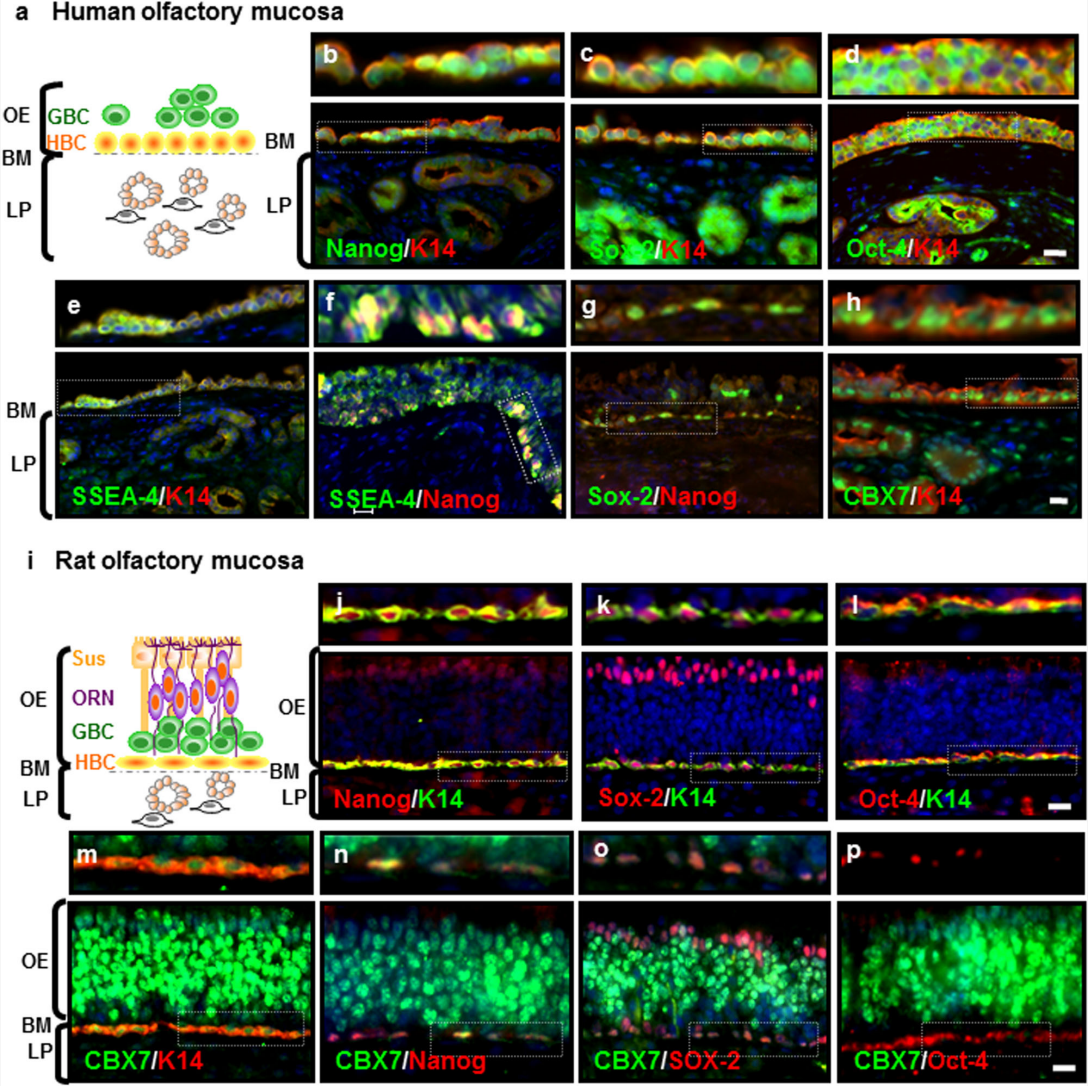
Olfactory basal membrane is the natural niche of APOSCs. **a–h** Adult human olfactory mucosa biopsy. A schematic of cell types, HBCs (major) and GBCs (seldom), remained in the human biopsy (**a**). Note that other cell types (*cf*. panel **I** for complete cell type composition) were lost during the sampling process. HBC resided directly adjacent to BM, which is indicated by the dotted line. LP is beneath the OE. In the adult human olfactory mucosa, double-label immunohistochemistry showed a cell population (putative APOSCs) coexpressing Nanog (**b**, green in cell nuclei), Sox-2 (**c**, green in cell nuclei), Oct-4 (**d**, green in nucleoli and cytoplasm), SSEA-4 (**e**, green on cell membrane), and CBX7 (**h**, green in cell nuclei) with K14 (red in cytoplasm). These basal layer-distributed cells also showed coexpression of either (**f**) SSEA-4 (green) and Nanog (red) or (**g**) Sox-2 (green) and Nanog (red). **i–p** Adult rat olfactory mucosa tissue. A schematic of major cell types in the adult rat olfactory mucosa (**i**). The pseudo-stratified OE is populated by Sus, ORNs, GBCs, and HBCs. In the rat biopsy, APOSCs were observed coexpressing Nanog (**j**, red), Sox-2 (**k**, red), Oct-4 (**l**, red), or Bmi-1 (**m**, red) with K14 (green). APOSCs also coexpressed CBX7 (green) with Nanog (**n**, red), Sox-2 (**o**, red), or Oct-4 (**p**, red). Cell nuclei were stained with DAPI (blue). OE olfactory epithelium, BM basal membrane, LP lamina propria, GBC globose basal cell, HBC horizontal basal cell, Sus sustentacular support cells, ORN olfactory receptor neuron. Scale bar, 20 μm

Next, we demonstrated the in vivo distribution of APOSCs. Double-label immunohistochemical analysis for human OM revealed that Nanog (Fig. 4b), Sox-2 (Fig. 4c), Oct-4 (Fig. 4d), and SSEA-4 (Fig. 4e) were all expressed by the cells distributed in the basal layer of OE. This putative APOSCs population coexpressed a basal-cell marker, cytokeratin 14 (K14, Fig. 4b–e). Further double-label immunohistochemistry confirmed their APOSCs identity because these basal cells coexpressed Nanog with either SSEA-4 (Fig. 4f) or Sox-2 (Fig. 4g). Note that in human OE, both HBC and GBC expressed K14 and had round cell bodies, unlike in murine OE where HBCs solely expressed K14 and displayed flat/horizontal morphology^31^. Moreover, coexpression of p63 and K14 by immunohistochemistry was also observed in HBC (Supplementary Fig. S4).

We next examined the murine complete olfactory mucosa, which was obtained from rat superior turbinate tissue. Similarly, only murine BM cells revealed coexpression of Nanog, Sox-2^32^, and Oct-4 with either K14 (Fig. 4j–l) or another basal-cell marker, ICAM-1 (intracellular adhesion molecule 1, data not shown). In line with these results, in vitro cultured APOSCs or spheres also expressed K14 (Supplementary Fig. S3a, b) or ICAM-1 protein (Supplementary Fig. S3c). Additionally, upon induced differentiation toward neurons, APOSCs lost expression of K14 but gained expression of Tuj-1 (Supplementary Fig. S3d).

### Necessity of CBX7 for APOSCs self-renewal

Furthermore, we explored the role of CBX7 as a putative mediator of the molecular mechanism that maintained APOSCs. First, CBX7 protein was expressed by human APOSCs or spheres in vitro (Supplementary Fig. S3e, f). Then, in adult olfactory tissues, CBX7 was expressed evidently in the nuclei of APOSCs, which were labeled with pluripotency-related markers Nanog (Fig. 4n), Sox-2 (Fig. 4o), and Oct-4 (Fig. 4p) or HBC marker K14 (Fig. 4h, m). Besides, CBX7 accumulated abundantly as polycomb-group bodies^33^ in ORNs (Fig. 4m), in agreement with previous reports that CBX7 is expressed in post-mitotic neurons of the mature brain and ocular tissues^34^. Aside from HBCs and ORNs, only a minimal level of CBX7 was observed in GBCs and sustentacular cells (Sus). Quantification of the specific population of HBC (CBX7^+^K14^+^), ORN (CBX7^+^NeuroD^+^), GBC (CBX7^+^Tuj-1^+^), and Sus (CBX7^+^K18^+^) by immunohistochemistry was 64 ± 11 cells/mm^2^, 295 ± 26 cells/mm^2^, 95 ± 1.2 cells/mm^2^, and 5 ± 3 cells/mm^2^, respectively (Supplementary Fig. S5).

**Fig. 5:**
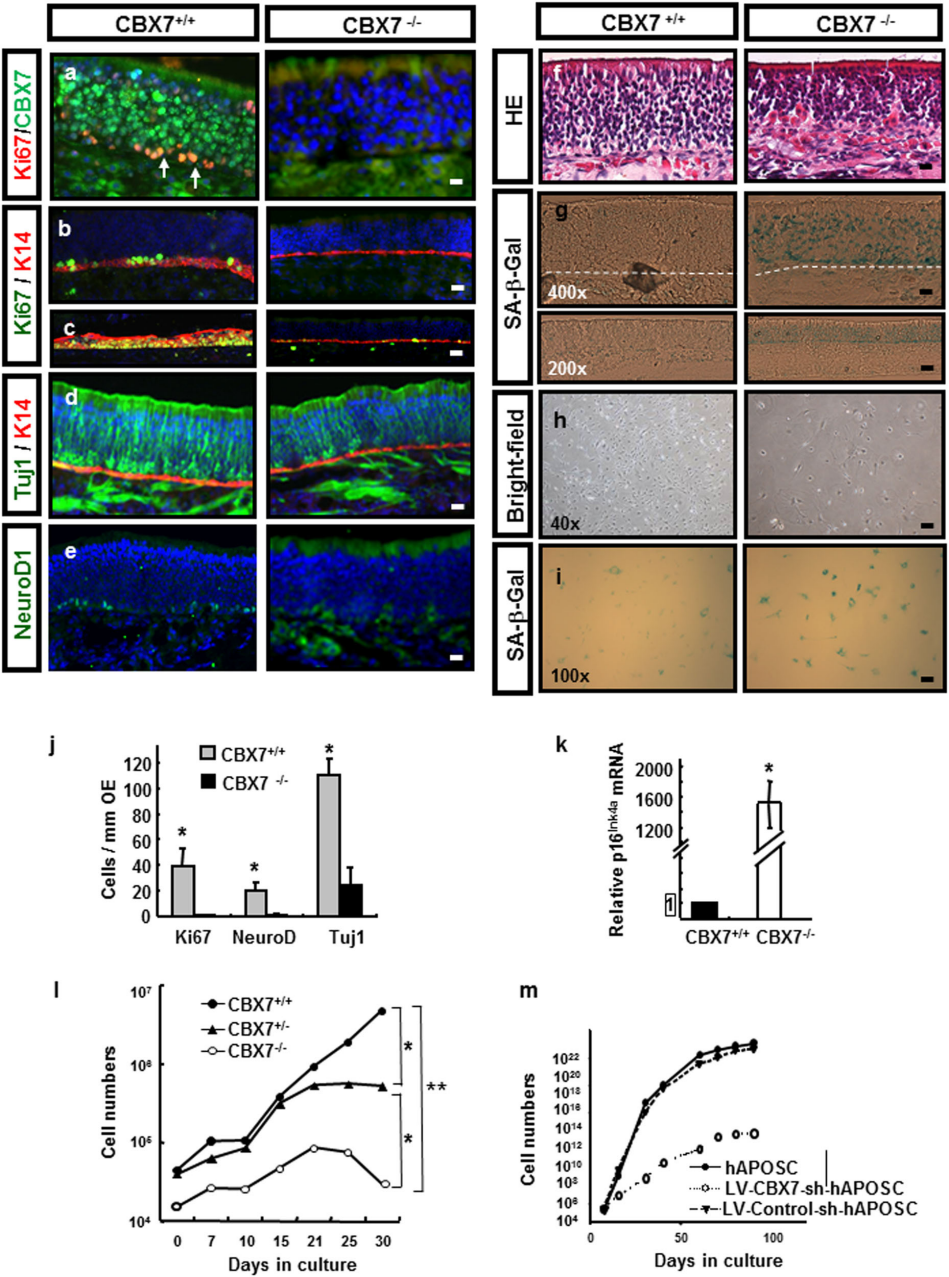
Self-renewal and antisenescence potential of APOSCs requires CBX7 expression. **a** The olfactory mucosa from CBX7^−/−^ showed no CBX7 expression (green) and decreased numbers of proliferating cells (Ki67, red) compared to the CBX7^+/+^ mice (Ki67, red, white arrow). **b**, **c** Uninjurbed (**b**) or methimazole-injured (**c**) olfactory mucosa tissues were examined for spontaneous or stimulated proliferation of APOSCs, respectively. Double-labeled immunohistochemistry for Ki67 (green) and K14 (red) revealed significantly reduced numbers of proliferating APOSCs in CBX7^−/−^ olfactory mucosa (right column), as compared with CBX7^+/+^ olfactory mucosa (left column). **d**, **e** Defective olfactory neuron differentiation in CBX7^−/−^ mice was indicated by reduced Tuj-1^+^ (**d**, green) and NeuroD1^+^ (**e**, green) cells. Cell nuclei were stained with DAPI (blue). Scale bar, 20 μm. **f** Hematoxylin/eosin (HE)-stained olfactory mucosa tissues. **g** Adult (8-weeks) olfactory mucosa tissue of CBX7^−/−^ mice showed SA-β-Gal activity (blue staining) in OE. Basal membrane is indicated by the dotted line. **h–i** Isolated- CBX7^−/−^ APOSCs exhibited flattened/enlarged morphology (**h**, right panel, on day 21) and SA-β-Gal activity (**i**, right panel, on day 32), as compared with the CBX7^+/+^ APOSCs (**h** or **i**, left panels, on day 21 or 32, respectively). **j** The percentages of Ki67-, NeuroD1-, Tuj-1-, and K14-expressing cells were quantitated in uninjured CBX7^+/+^ and CBX7^−/−^olfactory mucosa. **k** Quantitative RT-PCR analysis of p16^Ink4a^ in CBX7^+/+^ and CBX7^−/−^olfactory mucosa. **l** Proliferation arrest of CBX7^−/−^ mouse-derived APOSCs in culture. Cumulative cell numbers of mouse APOSCs derived from the indicated genotypes are shown. **m** ShRNA-mediated downregulation of CBX7 attenuated long-term expansion capacity of human APOSCs. OE: olfactory epithelium, SA-β-Gal: senescence-associated β-galactosidase. Data are presented as mean ± SD from two independent experiments of triplicate measurements; *P < 0.05, and **P < 0.01, Scale bar, 20 μm

Next, we found that some CBX7-expressing basal cells were actively proliferating (labeled with Ki67, Fig. 5a). The percentage of proliferating CBX7^+^Ki67^+^ cells was about 21.4 ± 0.3% of all CBX7-expressing basal cells. Therefore, we determined whether CBX7 was required for self-renewal of APOSCs within their natural niche. We monitored the proliferation of basal cells, as an indication for self-renewing APOSCs. In a steady state in CBX7^−/−^ mice, where CBX7 protein was not detectable in OE (Fig. 5a), only scarce spontaneous cell proliferation was observed among K14^+^-basal cells (Fig. 5b, j). In an injury-induced regeneration experiment, a chemical insult such as methimazole caused destruction of olfactory neurons; this treatment stimulated basal-cell proliferation and differentiation to replace the neuron loss^9^. At 3 days post injury, 79% of CBX7^+/+^ basal cells underwent proliferation, while only 32% CBX7^−/−^ basal cells could be stimulated to proliferate (Fig. 5c, as quantified by Ki67-positive cells among K14-positive cells). Accordingly, there were significant reductions in the numbers of immature sensory neurons (Tuj-1^+^ cells, Fig. 5d, j), committed neuronal precursors (NeuroD1^+^ cells, Fig. 5e, j), as well as morphology changes (Fig. 5f) in CBX7^−/−^ OE.

To test whether CBX7 played the same role in APOSCs, a senescence biomarker, β-galactosidase activity at pH 6, was analyzed in adult olfactory mucosa tissue. The apparent senescence-like phenotype revealed by blue staining was observed in CBX7^−/−^ APOSCs residing in the BM and olfactory neurons, whereas fewer blue-stained cells were detected in the CBX7^+/+^ olfactory mucosa tissue (Fig. 5g). Accordingly, the upregulation of p16^Ink4a^, which is involved in the induction of senescence, in CBX7^−/−^ olfactory mucosa was well pronounced (Fig. 5k). Next, we isolated APOSCs from CBX7^−/−^ mice and examined their phenotype. Similarly, in vitro-cultivated CBX7^−/−^ APOSCs showed premature senescence because they stop dividing after 21 days (Fig. 5l), acquired flattened/enlarged cell appearance (Fig. 5h), and 40% ± 5% cells showed abundant blue staining corresponding to senescence-associated β-galactosidase (SA-β-Gal) expression (Fig. 5i). In contrast, CBX7^+/+^ APOSCs kept dividing beyond 32 days (Fig. 5l), mostly retained spindle-shaped morphology (Fig. 5h) and only 16% ± 1% cells revealed SA-β-Gal expression (Fig. 5i). Similarly, when CBX7 in human APOSCs was downregulated by lentivirus short hairpin RNA (shRNA), the long-term expansion ability of hAPOSCs was significantly reduced (Fig. 5m). Besides, we did not detect increased apoptosis in CBX7^−/−^ olfactory mucosa tissue by Terminal deoxynucleotidyl transferase dUTP nick end labeling (TUNEL) assay (Supplementary Fig. S5b).

### A reduction in infarct size by intracerebral mAPOSC transplantation and improvement of neurological behavior after cerebral ischemia

**Fig. 6:**
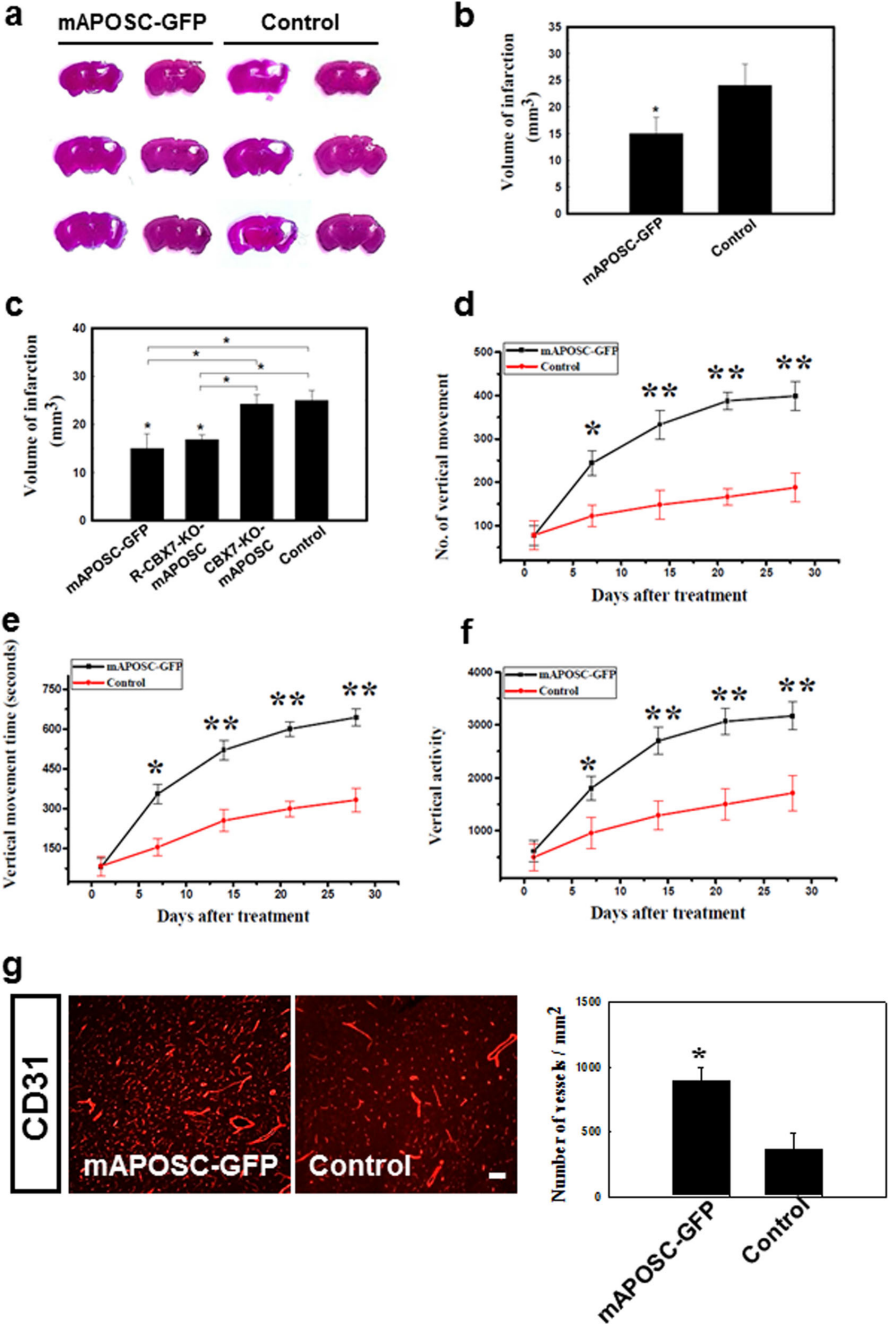
Mouse APOSCs transplantation improved neurological behavior in a stroke mouse model. **a**, **b** In representative brain slices stained with hematoxylin/eosin, a significant decrease in infarct volume was noted in the mAPOSC-GFP-treated mice as compared to the control one. **c** Significant reduction of infarct size was observed in stroke mice treated with stereotaxic injection of R-CBX7-KO-mAPOSC compared to that of CBX7-KO-mAPOSC-treated mice. **d–f** Motor functions were examined in mice receiving mAPOSC-GFP compared with vehicle control mice by means of locomotor activity presented as number of vertical movements (**d**), the vertical movement time (**e**), and vertical activity (**f**). **g** Quantitative analysis of blood vessel density showed more CD31^+^ vasculature in the mAPOSC-GFP-treated mice than in the control ones. *n* = 8 per group, Data are expressed as mean ± SEM; *P < 0.05

We next tested whether APOSCs hold a potential for stroke treatment. Stereotaxic mAPOSC-GFP transplantation into mice with stroke was performed to examine the infarct size and the neurological behavior function after cerebral ischemia. Greater reduction of infarct size was found in the mAPOSC-GFP-treated mice than in controls (Fig. 6a, b).

To demonstrate the rescuing effect of CBX7 overexpression, R-CBX7-KO-mAPOSC (1 × 10^6^ cells) were injected stereotaxically into stroke mice via three cortical areas for evaluating the infarct volume. Stereotaxic R-CBX7-KO-mAPOSC transplantation into mice showed significant reduction of infarct size compared to that of CBX7-KO-mAPOSC-treated mice (Fig. 6c).

Neurological behavior revealed significantly increased activity between 6 and 28 days after cerebral ischemia in mice receiving mAPOSCs transplantation, compared with control mice (Fig. 6d–f). To determine whether APOSCs implantation induced angiogenesis, blood vessel density was quantitatively measured by CD31 immunoreactivity. It revealed that in ischemic mice treated with APOSCs, the amount of neovasculature in the penumbric area significantly increased compared with that of controls (Fig. 6g).

### Pilot APOSCs implantation in stroke patients

To address the safety and feasibility of autologous APOSCs implantation for the clinical treatment of stroke-induced neurological dysfunction, we started recruiting patients in a pilot trial (http://clinicaltrials.gov identifier: #NCT01327768). Until now, according to follow-up of 12 months after implantation, six patients experienced no systemic or local adverse events (Table 1), thus providing preliminary evidence of the safety and feasibility of this therapeutic protocol. The complete results of further clinical trial will be the subject of a separate report.

**Table 1:** Clinical characteristics of recruited patients in pre-treatment stage

Item	Patient #1	Patient #2	Patient #3	Patient #4	Patient #5	Case#6
Age (years)	63 y/o	62 y/o	47 y/o	55 y./o	55 y/o	51 y/o
Men/Female	M	M	F	M	M	F
Duration of stroke (months)	58	11	13	16	32	14
Concomitant diseases:						
Hypertension	+	+	+	+	+	+
Diabetes	–	+	–	+	–	–
Hyperlipidemia	+	+	–	+	+	+
Atril fibrillation	–	–	–	+	–	–
Smoking	–	+	–	+	+	–
Prior aspirin or antiplatelet use	+	+	+	+	+	+
Rehabilitation duration (hours/week)	15	10	10	5	10	10
Baseline scores:						
NIHSS	11	10	10	9	10	10
ESS	46	44	34	34	46	46
EMS	18	24	35	29	26	16
FMT						
UE	48	38	74	61	61	58
LE	35	81	51	42	56	64
mRS	3	3	3	3	3	3

## Discussion

APOSCs, a new type of adult pluripotent-like stem cells, were found within both human and murine olfactory BM. Resembling ES cells, APOSCs exhibit characteristics including (i) positivity for pluripotency markers; (ii) ability to form floating spheres with self-renewal and ALP activity; (iii) effective differentiation into three layer cells both in vitro and in vivo. In contrast to ES cells, APOSCs display properties of being regulated by CBX7, which is distinctively essential for several other types of adult stem cells but not for ESCs;^35^ (ii) nontumorigenicity in immunodeficient mice; (iii) low telomerase activity and limited in vitro proliferation; (iv) no need for feeder layers for support of stem cell growth. Moreover, a pilot clinical trial involving autologous APOSCs implantation in stroke patients demonstrated the safety and feasibility of this therapeutic protocol (Table 2).

**Table 2:** Characteristics of APOSC implantation in each patient

Characteristics	Patient #1	Patient #2	Patient #3	Patient #4	Patient #5	Patient #6
A. S/S	Rt hemiparesis	Rt hemiparesis	Rt hemiparesis	Lt hemiparesis	Lt hemiparesis	Lt hemiparesis
B. MRI-T2WI						
C. Stroke history (months)	51	11	12	16	20	29
D. No. of injected cells	2 × 10^6^	2 × 10^6^	2 × 10^6^	2 × 10^6^	2 × 10^6^	2 × 10^6^
E. Purity of p75^+^ cells	91.5 %	90.4 %	95.5 %	91.7 %	91 %	91%
F. No. of IC trajectory	3	3	3	3	3	3

*S/S* symptoms and signs, MRI-T2* T2* image in MRI, *No.* numbers, *Lt* left limbs, *Rt* right limbs, *y* years, *IC* intracerrbral

In exploring the natural niche of APOSCs, their in vivo basal-membrane distribution was elucidated. Nonetheless, the specific relation between APOSCs and HBCs remains to be distinguished. Further studies should demonstrate the pluripotency of isolated HBC. HBC has been shown to exist in a tightly and loosely adherent phenotype. It is, therefore, suggested that a subpopulation of HBCs is responsible for its multipotent progenitor phenotype^36^. Whether APOSCs belong to the same HBC-subpopulation also should be studied. Besides, previous other studies showed that Sox-2 is expressed by olfactory basal cells^32^, but its function has not been characterized. The present study is the first demonstration that adult olfactory basal cells not only express other important ESCs markers, but also an adult stem cell gene, CBX7 (Table 3).

**Table 3:** Results of neurological scoring after OSC implantation

Scoring items	Baseline	6-month	12-month
NIHSS:	10.17 ± 0.37	7.5 ± 0.96**	6.0 ± 1.53**^,¶^
ESS:	41.67 ± 5.59	46.0 ± 1.63	48.0 ± 2.30*^,¶^
EMS:	24.50 ± 7.20	31.17 ± 7.82*	35.83 ± 8.27**^,¶¶^
FMT:			
UE	56.83 ± 11.31	75.33 ± 15.12*	88.67 ± 18.34**^,¶^
LE	57.50 ± 17.16	66.33 ± 16.18*	76.83 ± 17.70*^,¶^

*P < 0.05 and **P *<* 0.01 vs. baseline; ^¶^*P* < 0.05 and ^¶¶^*P* *<* 0^.^01 vs. 6-month

It is worth noting that APOSCs are distributed to the olfactory BM, where cells are normally quiescent, a hallmark of other adult tissue stem cells^37^. For example, recent studies show that the deeply dormant mouse HSCs have the highest self-renewal potential^38^. Interestingly, CBX7 is among a series of intracellular regulatory molecules that mediate the balance of dormant and self-renewing HSCs^16^. It is important to determine whether CBX7 is also responsible for switching the dormancy and self-renewal of APOSCs in their natural niche^15^. Moreover, CBX7 also marks a specific population of adult intestinal stem cells (ISCs) located at the base of intestinal crypts^39^. The CBX7^+^ ISCs are normally quiescent, whereas a second group of CBX7^low^ ISCs undergoes continuous proliferation^40^. This finding correlates with our observation that CBX7 is highly expressed in olfactory HBCs, yet GBCs expresses lower levels of CBX7. In the absence of CBX7, reduced self-renewal accompanied with premature senescence and p16^INK4a^ upregulation is evident in APOSCs. These data are consistent with other studies, which showed that CBX7 induced the cyclin-dependent kinase inhibitor (CDKI) p16^INK4a^ during senescence^41^.

In a stroke mouse model, the transplantation of APOSCs leads to migration of cells to ischemic sites; survival and differentiation into neuronal, glial, endothelial cell types; and functional improvement. Nonetheless, within 1 month after transplantation, the percentage of APOSCs differentiating into neurons is low. Whether their effective differentiation occurs at a later time point after implantation or APOSCs could serve as a source of trophic factors to attract endogenous stem cells for tissue-repair, needs further investigation.

Regarding the final result of this early clinical trial, we will prepare a complete full report in the other article. The preliminary findings revealed that magnetic resonance imaging (MRI)-DTI of the first patient, after comparison of before and 12 months after APOSCs treatment showed a prominent increase in the fiber numbers of cortico-spinal tract (CST) by fiber number asymmetry (FNA) scoring. Moreover, a simple linear correlation analysis was conducted to show the relation between clinical improvement and CBX7 expression in APOSCs. The percentage change of the Fugl-Meyer test (FMT) score measured at 12 months after cell implantation strongly correlated with the relative expression of CBX7. Patients with high expression of CBX7 in their autologous APOSCs seemed to present better clinical outcomes than those with low expression of CBX7 in their autologous APOSCs.

## Materials and methods

### Tissue preparation, animal strain, and primary cultures of APOSCs

Adult human Olfactory mucosa tissue (5 mm^3^, 0.5 g in weight, over the superior part of nasal cavity) was got from the nasal septum neighboring to the cribriform plate by a ethmoid forceps through the guidance of a nasal endoscope under general anesthesia^42,43^. Protocols for sampling adult human olfactory mucosa were approved by the Institutional Review Board of China Medical University and Hospital, Taichung, Taiwan. Written informed consent was obtained from all patients. For adult murine tissues preparation, Sprague-Dawley rats or mice, including C57BL/6 J Narl, GFP-transgenic mice^44^ were used. Animals of 8-week or 11-week-old were anesthetized, decapitated and their olfactory tissues (from superior turbinate) were isolated under a dissecting microscope.

Biopsy specimens of olfactory mucosa (three to five mucosal fragments) were collected into in sterile boxes containing Hanks’ balanced salt solution (Gibco/BRL) for primary culture within 24 h. In the explant culture method, the olfactory tissue was carefully dissected into small pieces under a dissecting microscope and placed in a phosphate-buffered solution at room temperature. The tissue explants were collected by centrifugation at 600 × *g* for 10 min. The resulting pellet was resuspended in DMEM/F12 medium containing 2 μg/mL heparin (Sigma), 20 ng/mL FGF-2 (R&D Systems) and 20 ng/mL EGF (R&D Systems) and 1% penicillin/streptomycin (P/S, 100 U/mL). The tissue explant was placed in a 25 cm^2^ flat flask and incubated in 5% CO_2_ at 37 °C. The tissue was left undisturbed for 5–7 days to allow for migration of the cells from the explants. These primary adherent cells called “adult pluripotent-like olfactory stem cells (APOSCs)” were passaged once reaching 80% confluence. For mouse APOSCs (mAPOSCs), the culture medium was further supplemented with 20 ng/mL EGF (invitrogen).

### Self-renewal assay and BrdU staining

Sub-confluency APOSCs were trypsinized and resuspended as 7 × 10^4^ cells per milliliter of sphere culture medium, consisting of DMEM/F12, 2% B27 supplement (Gibco), 20 ng/mL bFGF, 20 ng/mL EGF and 1% penicillin/streptomycin (100 U/mL). To avoid attachment of cells to the bottom of culture dishes, 15 mg/mL polyHEMA (Sigma, P3932) was coated on culture dishes before seeding the cell suspension. These primary sphere-forming cells arising from APOSCs were termed 1st APOSCs spheres.

Sphere numbers over multiple passages represent stem cells self-renewal activity, whereas sphere size demonstrates cell proliferation. To evaluate the self-renewal capacity of APOSCs spheres, 3 days-cultured 1st APOSCs spheres were trypsin-dissociated to single cells, cell number counted and re-plated in sphere culture medium. The subsequent spheres formed from trypsin-dissociated 1st spheres were termed 2nd spheres. For growth detection, the diameters of 2nd APOSCs spheres were measured at day 2, 5, and 9 of cultures. For BrdU labeling and staining, spheres were cultivated in the presence of 10 µM BrdU (Sigma) for 24 h and seeded onto poly-d-lysine-coated wells for 1 h. Cells were fixed with 4% PFA for 10 min and washed with PBS. Fixed cells were incubated in 1 N HCl for 10 minutes on ice followed by incubation in 2 N HCl for 1 h at 37 °C, neutralization in 0.1 M borate buffer, permeabilizing in 1% Triton-X-100 for 1 h, washed and blocking in 5% FBS for 1 h. Mouse-anti-BrdU antibody (1:1000, Sigma) was incubated with cells at 4 °C overnight. FITC-anti-mouse antibody was then used and followed by 1 h of PI (1:1000) staining.

### CSFE-labeling and cell proliferation analysis

To monitor cell proliferation, APOSCs were pulse labeled with 10 M CFSE (carboxyfluorescein succinimidyl ester, Molecular probes). After 4 days of culture, the CFSE staining dilution profile (indicating cell proliferation) was evaluated by flow cytometry (Becton Dickinson). The data was calculated using MODFIT software (Verity Software House, Topsham, ME).

### Gene silencing with RNA interference

Specific knockdown was achieved by lentiviral delivery of shRNA for CBX7 (LV-CBX7-sh; sc-72816-V, Santa Cruz Biotechnology), and the control shRNA (LV-control-sh; sc-108080, Santa Cruz Biotechnology) under manufacturer’s instruction.

### Immunohistochemistry and immunocytochemistry

For Immunohistochemistry, olfactory mucosa tissues harvested from adult human or rat were fixed in 4% paraformaldehyde, dehydrated in 30% sucrose, cryoprotected, and frozen in OCT (Sakura Finetek). Cryosections of 10 μm were cut, stained with H&E and observed by light microscopy (Nikon, E600, Japan) as well as stored at –80 °C until needed. For Immunocytochemistry, cells were fixed in 4% paraformaldehyde for 10 minutes and washed with PBS. Thereafter, tissue sections or cells were permeabilized with 0.3% Triton-X-100 for 30 min, washed with PBS-T (0.1% Tween-20 in PBS) and incubated with blocking solution (5% FBS in PBS) for 1 h. Then samples were incubated overnight at 4 °C with primary antibodies: Oct-4 (1:100, Abcam), Nanog (1:200, Navous), Sox-2 (1:100, Cell signaling), SSEA-4 (1:100, Millipore), K14 (1:250, GeneTex), CBX7 (1:100, Millipore), p63 (1:100, Millipore), K18 (1:300, Millipore), NeuroD1 (1:500, Abcam), Tuj-1 (1:1500, Covance), MAP2 (1:500, Millipore), GFAP (1:300, Millipore), O4 (1:1000, Millipore), CD45 (1:200, R&D), and Ki67 (1:200, Thurmo). Samples were then incubated with Alexa-488 (or-594)-conjugated anti-rabbit (or anti-mouse) IgG antibodies (Invitrogen), followed by mounting with Dapi Fluoromount-G (SouthernBiotech). Images of samples were taken with a fluorescence microscope (Nicon TS100). The cell numbers quantification of each specific population of HBC (CBX7^+^K14^+^), ORN (CBX7^+^NeuroD^+^), GBC (CBX7^+^Tuj-1^+^) and Sus (CBX7^+^K18^+^) was examined by counting the double positive cells over ten high-magnification view per mm^2^ using NIH ImageJ.

Cells stained for alkaline phosphatase were fixed with 80% ethanol (4 °C, overnight), washed with distilled water, incubated with 100 mM Tris-HCl (pH8) and stained using Vector Red Alkaline phosphatase Substrate Kit I according to the manufacturer’s instructions.

### Flow cytometry

APOSCs were dissociated by 0.25% trypsin-EDTA, washed with PBS containing 2% BSA, and incubated with primary antibodies for SSEA-4 (1: 50, Millipore), SSEA-3 (PE-conjugated, BD), or ICAM-1 (1:100, GeneTex). For intracellular proteins, cells were fixed and permeabilized using a cell staining kit (eBiocience 88-8115) before applying primary antibodies, Oct-4 (1:100, Abcam), Nanog (1:100, GeneTex), and Sox-2 (1:50, Millipore). Cells were then detected by anti-mouse IgG, anti-rabbit IgG or anti-rat IgM conjugated with FITC. FITC-conjugated IgG2a antibodies were used as isotype controls. Thereafter, the cells were analyzed using a FACSTAR^+^ flow cytometer (Becton Dickinson).

### Quantitative RT-PCR

Total cellular RNA was isolated using TRIzol reagent and then digested with DNase I to remove genomic DNA. The cDNA was generated using SuperScript III first-strand synthesis SuperMix kit (Invitrogen). The PCR reaction was performed using Dream Taq DNA polymerase (Fermentas). For detecting stem cell pluripotency markers, Oct-4, Sox-2, Nanog, c-Myc, CBX7 and KLF-4, the primers reported previously^23,45^ were used. For detecting tissue specific differentiation gene expression, the primers reported by Dezawa^46^ were used. Quantitative PCR was performed on an Applied Biosystems instrument using FastStart Universal SYBR Green Master (Roche). The p16^Ink4a^ primers reported by Molofsky^28^ were used.

### Induced and spontaneous APOSCs differentiation in vitro

For neuron induction, APOSCs were cultured in poly-d-lysine-coated wells with DMEM/F12 medium containing 25 ng/mL BDNF (Peprotech), 1% N2 supplement (Gibco), 50 ng/mL brain-derived neurotrophic factor (BDNF, Peprotech). After 10–14 days, cells were subjected to immunocytochemistry for detecting neuron-specific genes, Tuj-1 (1:1500, Covance), MAP2 (1:500, Millipore), astrocyte-specific gene of GFAP (1:300, Millipore), and oligodendrocyte marker of O4 (1:1000, Millipore) expression.

Adipocyte, chondrocyte, and osteocyte inductions were performed according to STEMPRO adipogenesis, chondrogenesis, and osteogenesis differentiation kits (Gibco) respectively. Adipogenic differentiation of APOSCs could be identified via Oil Red O staining (0.3% oil red O (Sigma) for 15 min and washed with water) as an indicator for intracellular lipid accumulation. Osteogenesis via Alizarin Red S staining was used to detect calcium mineralization. Chondrogenesis via Alcian Blue staining could indicate proteoglycans synthesized by chondrocytes generated from APOSCs. Alcian Blue or Alizarin Red S stain analyses were performed according to manufacturer’s protocol.

To induce APOSCs toward endothelial cells, cells were grown in DMEM medium supplemented with 20% FBS, 50 ng/mL VEGF, 25 ng/mL bFGF and 5 units/mL heparin. After 8–10 days, cells were analyzed by in vitro tube formation assay or immunocytochemistry to determine endothelial cells. For in vitro tube formation assay, Matrigel (BD Biosciences) was twofold diluted with PBS and added to wells of a 24-well plate in a volume of 0.289 mL. Matrigel was allowed to solidify at 37 °C for 30 min. Differentiated (VEGF-induced) APOSCs (4.8 × 10^5^ cells) were resuspended in 0.3 mL of EGM-2 medium (Lonza) and added into Matrigel-coated wells. The tube networks were imaged using a microscope (Nikon TS100).

For hepatocyte induction, APOSCs (4 × 10^4^ cells/2-cm^2^) were grown on collagen I- or Matrigel (1%)-coated wells for 14 days in DMEM (low glucose) medium containing 50 ng/mL hepatocyte growth factor (HGF, Peprotech), 50 ng/mL FGF-4 (Peprotech), 1 × insulin-transferein-selenium (ITS, Gibco) and 10 nM dexamethasone (Sigma). In additional 5 days, culture medium was replaced with hepatotic commitment medium:^47^ Iscove’s modified Dulbecco’s medium (IMDM) supplemented with 20 ng/mL oncostatin M (Peprotech), 0.4 µM dexamethasone and 1 × ITS. Next, APOSCs were subjected to immunocytochemistry for detecting hepatocyte-specific genes, including Albumin (1:100, Bethyl), α1-anti-trypsin (1:200, Thermo) and APF (1:100, Daco). To test for hepatocellular metabolic functions, polyglycans were stained by a PAS method, indicating glycogen storage within the cytoplasm of differentiated cells. The PAS stain kit (ScyTek Laboratories) was used. Shortly, fixed (4% PFA, 10 min) cells were oxidized in periodic acid for 10 min, rinsed in PBS and treated with Schiff’s reagent for 30 min. Afterward, cells were rinsed in PBS and counterstained with hematoxylin.

For spontaneous differentiation, APOSCs were grown on gelatin (0.1%, G1890, Sigma)-coated culture wells with DMEM containing 20% FBS. After 14 days, cells were subjected to immunocytochemistry to determine whether APOSCs express specific markers for neuron (Tuj-1 and MAP2) and astrocyte (GFAP), muscle (SMA, 1:100, Abcam) or hepatocyte (albumin, α1-anti-trypsin, glycogen deposition). In addition, RT-PCR was used to detect tissue specific differentiation gene products.

### Olfactory neuroepithelium lesion

Methimazole (Sigma) was diluted at 5 µg/µL in PBS and injected intraperitoneally to mice at 50 mg/Kg of animal weight. Olfactory tissue was fixed at 3 days following methimazole injection.

### Telomerase activity assay

Telomerase activity was analyzed using telomere repeat amplification protocol (TRAP) by using the TeloTAGGG telomerase PCR ELISA kit according to manufacturer’s instructions (Roche). In brief, telomerase activity extends telomeric repeats (T2AG3) to the terminal end of biotin-labeled primers. The extension products of telomerase were amplified using PCR. The PCR product by telomerase extension was examined via hybridization to digoxigenin-labeled probes. The level of enzyme activity was evaluated and determined by photometric enzyme immunoassay.

### Senescence-associated (SA) β-galactosidase (SA-β-Gal) assay

SA-β-Gal staining was detectable at pH 6 as described previously^48^. Cells were fixed with 0.2% glutaraldehyde and 4% paraformaldehyde for 3 min. Tissues were fixed with 4% paraformaldehyde. After three PBS washes, cells or tissues were incubated at 37 °C (no CO_2_) overnight with fresh stain solution: 1 mg/mL X-gal, 5 mM potassium ferricyanide, 5 mM potassium ferrocyanide, 2 mM MgCl_2_, 150 mM NaCl, in citrate/sodium phosphate buffer (40 mM, pH 6). Images of samples were captured with a microscope (Nikon TS100).

### CBX7 knockout mouse lines

The colony of each mouse line was maintained in the animal facility of the China Medical University, Taiwan according to the Institutional Ethical Committee for Animal Research. For generation of knockout (KO) mice, according to the design principle and program of CRISPR/Cas9, we designed two target sgRNAs (CBX7T1 and CBX7T2, Taiwan Transgenic Mouse Core Facility), which respectively target exon 5 and exon 6 of CBX7 in mouse genome to delete the fifth and sixth codon of the mouse *Cbx7* gene as previously described^49^. In order to detect the off-target mutations, genome-wide, unbiased identification of DSBs (GUIDE-seq) based on global capturing of DSBs introduced by RNA guided endonucleases (RGEN) was applied to enable genome-wide profiling of off-target cleavage by CRISPR-Cas nucleases (Thermo Fisher) to improve the off-target effect^50^. The homozygote normal littermate (NL) and heterozygote newborns (*CBX7*^*+/–*^) were genotyped according to the procedure established by PCR using primers as described^49^. *CBX7*^*+/–*^ mice were crossed with each other to yield *CBX7*^*–/–*^ animals (CBX7-KO). The Institutional Ethical Committee for Animal Research at China Medical University has reviewed and approved all animal experiments.

### Teratoma formation in vivo

APOSCs (1 × 10^6^ cells in 100 mL) were resuspended in DMEM containing 10% FBS and was injected subcutaneously into both dorsal flanks of nude mice (CByJ.Cg-Foxn1nu/J) anaesthetized with isoflurane. Six weeks after injection, teratomas mass was dissected, fixed overnight in 10% buffered formalin phosphate and embedded in paraffin. Sections were stained with haematoxylin and eosin.

### Transplantation of APOSCs into stroke mice

Adult male C57BL/6 mice (25–30 g) were subjected to two-vessel ligation. All surgical procedures were performed by sterile/aseptic techniques in accordance with Institutional Guidelines for Animal Research. Rats were anesthetized with chloral hydrate (0.4 g/kg, i.p.). Ligations of the right middle cerebral artery (MCA) and right common carotids arteries (CCAs) were performed as described previously^51^. The right CCAs were clamped with non-traumatic arterial clips. Using a surgical microscope, a 2 × 2 mm craniotomy was drilled where the zygoma fuses to the squamosal bone. The right MCA was ligated with a l0-0 nylon suture. Cortical blood flow (CBF) was measured continuously with a laser Doppler flowmeter (PF-5010, Periflux system, Sweden) in anesthetized animals. A 1-mm burr hole was made in the right fronto-parietal region to place photodetectors. A probe (0.45 mm in diameter) was stereotaxically placed in the cortex (l.3 mm posterior, 2.8 mm lateral to the bregma, and l.0 mm below the dura). After 120 min ischemia, the suture on the MCA and the arterial clips on CCAs were removed to allow reperfusion. During anesthesia, core body temperature was monitored with a thermistor probe and maintained at 37 °C using a heating pad. After recovery, body temperature was maintained at 37 °C with a heat lamp^52^.

Prior to cells transplantation, human APOSCs (hAPOSCs) were first transduced with a lentivirus encoding the *Luc* gene as described previously^53^, which contributed to luciferase expression on the cells (Luc-UMSCs). For intracerebral cell transplantation, mice (C57BL/6 J Narl) were subjected to an ischemia/reperfusion stroke model as described previously^44^. The mouse under cerebral ischemia was injected stereotaxically with 10^6^ cells of hAPOSC-Luc into three cortical areas, 3.5 mm below the dura. For liver transplantation of neonatal (day 2) mice, 10^6^ cells of hAPOSC-Luc were transcutaneously injected into the liver with a 31-gauge syringe^30^. Cyclosporin A (CsA; 1 mg/kg/day, i.p.; Novartis) injections were given daily to each experimental rat from the day after cerebral ischemia for 3 weeks^54^. At 4 weeks (after brain-transplantation) and 6 weeks (after liver-transplantation), mice were subjected to immunohistochemistry.

Moreover, CBX7^−/−^ mAPOSCs (CBX7-KO-mAPOSC) was transduced with the particle (1 × 10^8^) of pLenti-C-CBX7-mGFP (Origin) to build as the R-CBX7-KO-mAPOSC for inducing CBX7 overexpression. Then, the stroke mice were injected stereotaxically with 10^6^ cells of R-CBX7-KO-mAPOSC into three cortical areas for evaluating the infarct volume.

### Infarct volume measurement

To analysis the infarcted brain, a series of 2-mm thick coronal sections with a 200-µm interval were cut by a cryostat. Tissue sections were stained with H&E staining (Sigma) at room temperature. To measure the infarct area in the right cortex, we subtracted the noninfarcted area in the right cortex from the total cortical area of the left hemisphere. The area of infarct was drawn manually from slice to slice, and the volume was then calculated by internal volume analysis software (NIH ImageJ)^55^.

### Neurological behavioral measurement

Behavioral assessments were performed before and after cerebral ischemia in mice. The motor functions were tested by locomotor activity and rotarod measurement. Mice were subjected to VersaMax Animal Activity monitoring (Accuscan Instruments, Columbus, OH) for 2 h for behavioral recording of each of the experimental mice. The VersaMax Animal Activity monitor contained 16 horizontal and 8 vertical infrared sensors spaced 87 cm apart. The vertical sensors were situated 8 cm from the floor of the chamber. Locomotor activity was counted as the number of beams broken by a rat’s movement in the chamber. Three vertical parameters defined in the manufacturer’s menu option were calculated over 2 h: vertical activity, vertical time, and number of vertical movements.

### Terminal deoxynucleotidyl transferase dUTP nick end labeling (TUNEL) cytochemistry

Cellular apoptosis was assayed by immunohistochemistry using a commercial TUNEL staining kit (DeadEnd Fluorimetric TUNEL system; Promega) as previously described^56^. The percentage of TUNEL labeling was expressed as the number of TUNEL-positive nuclei divided by the total number of nuclei stained with DAPI^57^.

### Evaluation of angiogenesis

To quantify the cerebral blood vessel density, histological mice brain sections (6 μm) were stained with specific antibody to CD31 (1:100; BD Biosciences)-conjugated with cyanine-3 (1:500; Jackson ImmunoResearch Laboratories), and the number of blood vessels were determined as described previously^58^.

### Clinical trial for human subjects

This trial was approved by the Institutional Review Board (IRB) of the China Medical University Hospital, Taichung, Taiwan and registered at the ClinicalTrials.gov website (#NCT01327768). Six patients were enrolled in the trial under the approval of Taiwan Food and Drug Administration (TFDA). Autologous APOSCs were isolated and expanded as described above in the context of a quality management system and Good Tissue Practice (GTP) facility established within the China Medical University Hospital. During transplantation, a stereotaxic frame (LEKSELL model G, USA) for targeting the brain with MRI guided-computerized software (BrainLab, USA) was placed on the patients’ head under local anesthesia. After a burr hole-craniotomy was created, the viable APOSCs (2 × 10^6^) through a 1.5-mm inner diameter needle (LEKSELL Stereotaxic System, USA) equipped with Hamilton syringe was stereotaxically inserted down to the deepest target point as the implantation site. MRI protocol for processing the diffusion tensor image (MRI-DTI) data^59^ was performed using a 3-Tesla magnetic resonance imager (GE) with sagittal T2-weighted fast spin echo sequence. All patients were followed every 1 to 3 months in the clinic for 12 months. MRI-DTI data were obtained to measure the fiber numbers as fiber number asymmetry (FNA) score as previously described^59,60^. An independent safety committee monitored the results of the trial including frequency of adverse reaction (AEs). The primary end points evaluated by clinical scoring of Fugl-Meyer test (FMT) were determined at baseline, 6 and 12 months after APOSCs implantation.

### Statistical analysis

All measurements in this study were performed in a blinded design. Results were expressed as mean ± SEM. The behavioral scores were evaluated and adjusted by normal distribution. Differences between groups were evaluated by two-way ANOVA with the Newman-Keuls post hoc test. A simple linear correlation analysis was used to investigate the relationship between relative expression of CBX7 of each APOSCs and FMT scores relative to baseline. The correlations between these variables were determined by the Pearson correlation coefficient. The differences of FNA scores at three time points (at baseline, 6 months, and 12 months) in each patient was performed with Bonferroni corrected one-way ANOVA. *P*-value < 0.05 was considered significant.

## Electronic supplementary material

Potential role of CBX7 in regulating pluripotency of adult human pluripotent-like olfactory stem cells in stroke model
