# Supplementary material for: Potential role of CBX7 in regulating pluripotency of adult human pluripotent-like olfactory stem cells in stroke model
Source: Cell Death Dis. 2018 May 2;9(5):502. doi: 10.1038/s41419-018-0519-8 (PMC5931587; doi:10.1038/s41419-018-0519-8)
Supplement: Supplementary file 1 — Potential role of CBX7 in regulating pluripotency of adult human pluripotent-like olfactory stem cells in stroke model [file 41419_2018_519_MOESM1_ESM.doc]

**Potential role of CBX7 in regulating pluripotency of adult human pluripotent-like olfactory stem cells in stroke model**

Jia-Rong Fan^1*^, Hsu-Tung Lee^2*^, Wei Lee^1^, Chen-Huan Lin^1^, Chun Y. Hsu^3^, Chia-Hung Hsieh^3^ and Woei-Cherng Shyu^1,3,4^

**Supplemental Information**

**Figure S1. Characterization of mouse APOSCs by immunocytochemistry (ICC).** Isolated mouse APOSCs could grow as an adherent monolayer (a-c) or as floating spheres (d-g). Immunocytochemical (ICC) analysis showed that mouse APOSCs expressed Nanog (a and g), Oct-4 (b and f), and Sox-2 (c). Cell nuclei were stained with DAPI (blue). (d) Ki67 (green) and DAPI (blue) staining of a primary APOSCs sphere. (e) ALP staining of a primary APOSCs sphere. (h) RT-PCR analyses for pluripotency-related genes transcribed in mouse APOSCs. Mouse ES (mES) cells served as the positive control (10-fold diluent cDNA). Templates without reverse transcription served as the negative control (-). (i) ICC staining of CD45 (green) and DAPI (blue) as the negative control. (j) No immunoreactivity in non-differentiated APOSCs was noted in the representative differentiation markers of three germ layers (Ectoderm: GFAP; Mesoderm: α-SMA; and endoderm: albumin). (k) APOSCs (both CBX7^+/+^ and Cbx7^-/-^ cells) showed lower telomerase activity than that of ESCs. (l) Prominent teratoma formation (black arrow) was found in ESCs inoculation to nude mice (right panel), but there was no teratoma mass after the human APOSCs inoculation (left panel). Data are presented as mean ± SD from two independent experiments of triplicate measurements; **P* < 0.05, and ***P* < 0.01, Scale bar, 20 μm.

**Figure S2. In vivo differentiation of mouse APOSCs in the ischemic mouse brain.** **(a-d)** Intracerebrally transplanted mAPOSC-GFP (GFP^+^, green) expressed MAP2 (A, red), Nestin (B, red), vWF (C, red), and laminin (D, red). Cell nuclei were stained with DAPI (blue). Scale bar, 20 m.

**Figure S3. Expression of HBC markers and CBX7 in isolated APOSCs. (a and b)** Immunocytochemistry of K14 in isolated human APOSCs (A, green) or APOSC-formed spheres (B, green). **(c)** Flow cytometric analysis of isolated human APOSCs for ICMA-1 expression (open area curve). Proportions of positive cells (%) are shown in the upper right of the panel as compared to control (filled area curve). **(d)** 11 days after induced differentiation toward neurons, some APOSCs expressed Tuj-1 (green, arrow) but not K14 (red) as revealed by immunocytochemical analysis. **(e and f)** Immunocytochemical analysis of CBX7 (green) in isolated human APOSCs (E) or APOSC-formed spheres (F). Cell nuclei were stained with DAPI (blue). Scale bar, 20 m.

**Fig. S4.** Co-expression of K14 (green) and p63 (red) in HBCs of isolated human APOSCs (K14^+^p63^+^ cells). Cartoon figure (left panel) was showed to indicate the location of the each cells’ population. Sustentacular cells (white arrows) was observed in the superficial layer of olfactory epithelium. Scale bar, 20 m.

**Fig. S5. CBX7 is required for APOSCs self-renewal in OE.** (a) Representative bar graph was showed for quantification of specific population of OE including HBC (CBX7^+^K14^+^), ORN (CBX7^+^NeuroD^+^), GBC (CBX7^+^Tuj-1^+^) and Sus (CBX7^+^K18^+^) by immunohistochemistry. (b) Lack of CBX7 level did not increase the cellular apoptosis in the CBX7^-/-^ OE in comparison to that of CBX7^+/+^ OE by TUNEL assay (ischemic brain as TUNEL positive control). Scale bar, 20 m.
